# Safety Analysis of Simultaneous Vaccination of Japanese Encephalitis Attenuated Live Vaccine and Measles, Mumps, and Rubella Combined Attenuated Live Vaccine from 2020 to 2023 in Guangzhou, China

**DOI:** 10.3390/vaccines13040417

**Published:** 2025-04-16

**Authors:** Jie Liu, Yong Huang, Fengrui Jing, Yan Kang, Qiaojuan Liu, Zhiwei Zheng, Chunhuan Zhang, Xiaofeng Liang, Zhoubin Zhang

**Affiliations:** 1 School of Public Health, Southern Medical University, Guangzhou 510515, China; liujie2621819568@163.com; 2 Department of Immunization Programme Planning, Guangzhou Center for Disease Control and Prevention, Guangzhou 510440, China; gzcdc_huangy@gz.gov.cn (Y.H.); gzcdc_kangy@gz.gov.cn (Y.K.); gzcdc_liuqj@gz.gov.cn (Q.L.); zhengzhw5@mail2.sysu.edu.cn (Z.Z.); gzcdc_zhangch@gz.gov.cn (C.Z.); 3 Department of Public Health and Preventive Medicine, School of Medicine, Jinan University, Guangzhou 510632, China; jingfengrui@jnu.edu.cn; 4 Guangdong Provincial Institute of Public Health, Guangdong Provincial Center for Disease Control and Prevention, Guangzhou 511430, China; 5 Department of Communicable Disease Control and Prevention, Guangzhou Center for Disease Control and Prevention, Guangzhou 510440, China

**Keywords:** Japanese encephalitis attenuated live vaccine, Measles-mumps-rubella vaccine, measles, mumps, and rubella combined attenuated live vaccine, simultaneous vaccination, safety, adverse reaction

## Abstract

Objectives: Our objectives were to evaluate the safety of the simultaneous vaccination of Japanese encephalitis attenuated live vaccine (JEV-L) and measles, mumps, and rubella combined attenuated live vaccine (MMR) in children and to provide a reference for the implementation of the strategy of simultaneous vaccination with the two vaccines. Methods: The data of adverse events following immunization (AEFI) and vaccination for JEV-L and MMR from 2020 to 2023 were extracted through the Guangdong Province Vaccine Distribution and Vaccination Management Information System and the Chinese National AEFI Information System (CNAEFIS). The inclusion criteria were that children were born after 1 October 2019, and received the first dose of JEV-L or MMR after 1 June 2020, in accordance with the starting age for vaccination (8 months). The study used the number of vaccine doses as the denominator to calculate and compare the reporting rates of cases and calculated the relative risk (RR) of adverse reactions and the 95% confidence interval (CI). Results: In Guangzhou, a total of 214,238 doses of JEV-L were administered to children. JEV-L and MMR were co-administered in 464,009 doses, and MMR was administered separately in 241,150 doses. The overall reporting incidence rates of AEFI (per 100,000 doses) for JEV-L, the simultaneous vaccination group, and MMR were 11.20, 53.02, and 60.96, respectively. Among children aged 8 months in Guangzhou, 57.98% (463,512/799,423) received the simultaneous administration of JEV-L and MMR. In the reported AEFI events, general reactions accounted for 87.50% in the JEV-L group, 88.21% in the simultaneous vaccination group, and 89.80% in the MMR separate group. The incidence rates of common adverse reactions were 9.80, 46.7, and 54.74, respectively. The incidence rates of rare adverse reactions were 0.93, 3.88, and 2.90, respectively. The reporting incidence rates of fever ≥38.6 °C after vaccination were 4.20, 16.16, and 17.83 for the JEV-L separate group, simultaneous vaccination group, and MMR separate group, respectively. There was a significant difference between the simultaneous vaccination group and the JEV-L separate group (RR = 3.848, 95% CI = 1.927, 7.683), while no significant difference was found compared with the MMR separate group (RR = 0.906, 95% CI = 0.623, 1.318). The simultaneous vaccination group showed no significant differences in the reporting incidence rates of local redness and induration compared with the two separate vaccination groups (RR = 1.385, 95% CI = 0.144, 13.315; RR = 0.390, 95% CI = 0.087, 1.743; RR = 0.520, 95% CI = 0.033, 8.314). No significant differences were found in the incidence rates of rare adverse reactions such as maculopapular rash, urticaria, and thrombocytopenic purpura. Conclusions: The AEFI reporting incidence rate for the first dose of the simultaneous vaccination of JEV-L and MMR in 8-month-old children in Guangzhou is between the rates of the two separate groups. Compared with the MMR separate group, the simultaneous vaccination group does not increase the risk of adverse reactions.

## 1. Introduction

Vaccination is the most cost-effective and efficient measure for preventing infectious diseases, and it is a milestone in mankind’s fight against infectious diseases. Japanese encephalitis virus is the main cause of viral encephalitis in many countries of Asia, with an estimated 100,000 clinical cases every year [1]. The SA14-14-2 strain of Japanese encephalitis attenuated live vaccine (JEV-L) is an original creation of China that not only has high protection efficiency but also has good safety and a low incidence of adverse reactions [2]. Since 2008, the JEV-L has been included in China’s National Immunization Program (NIP). The incidence and case fatality rate of Japanese encephalitis have significantly decreased nationwide [3], reaching 0.0145 per 100,000 population by 2023 [4].

Measles, mumps, and rubella are respiratory diseases caused by the measles virus, mumps virus, and rubella virus, respectively. Since China included the measles, mumps, and rubella combined attenuated live vaccine (MMR) in the NIP in 2008, the national reported incidence of measles has decreased year by year from 2015 to 2022 [5,6]. However, in 2023, there was a resurgence of measles in China, with an incidence rate of 0.0441 per 100,000 [4]. Meanwhile, globally, the incidence of measles rebounded to 28.8 per 1 million in 2022 [7], indicating that neither China nor the World Health Organization (WHO) has yet achieved the goal of eliminating measles. The incidence of rubella in China has decreased year by year since 2010, but it rebounded significantly in 2018 [5]; compared with the period before the inclusion in the NIP, there was no significant change in the reported incidence rate of mumps [8]. From 2008 to 2022, the average annual incidence rate of mumps in Guangzhou was 26.08 per 100,000. The incidence rate showed a downward trend from 2014 to 2022, with the rate reaching 6.90 per 100,000 in 2022 [9]. This decline may be related to the free administration of the first dose of the MMR vaccine during this period [10]. However, the incidence rate in Guangzhou remains higher than in regions such as Beijing and Tianjin, which have implemented a two-dose MMR vaccination schedule [11,12].

In order to further control the spread of infectious diseases, starting from 1 June 2020, China adjusted the vaccination of 8-month-old children with measles and rubella combined attenuated live vaccine (MR) to MMR, and in accordance with the immunization program for children with NIP vaccines, they can be vaccinated with JEV-L or the Japanese encephalitis inactivated vaccine at the same time [13].

Simultaneous administration refers to the practice of administering two or more vaccines to the same individual on the same calendar day, at different anatomic sites or by different routes of administration, and not by mixing the vaccines in the same syringe before injection [14,15]. Surveys have shown that in Guangdong Province, after the implementation of simultaneous vaccination, the number of clinic visits for children under 2 years old was reduced from the original 25 to 14 [16]. This has saved costs related to multiple trips to the vaccination clinics, including vaccination fees, time off work, and transportation costs. The experience from Italy indicates that the simultaneous administration of multiple vaccines can increase the coverage rate of childhood vaccinations [17]. Kenya has also clarified that simultaneous vaccination is significantly associated with timely vaccination [18]. Domestic and international studies on the safety of the simultaneous administration of vaccines containing measles components, JEV-L, and any other vaccine have found that adverse reactions are generally mild and show no significant difference in incidence compared with those of single-dose vaccination [19,20,21,22,23,24,25]. In clinical trials of simultaneous administration of JEV-L and MMR vaccines, studies in Taiwan [26], China [27], and the Philippines [28] have shown that the simultaneous administration of these two vaccines in children aged 8 to 12 months is non-inferior to the administration of either vaccine alone. It does not adversely affect safety or reactogenicity. In addition, Shandong Province has shown that the adverse reaction reporting rates (per 100,000 doses, the same below) for the simultaneous administration of JEV-L and MMR, MMR alone, and JEV-L alone were 107.09, 123.60, and 49.79, respectively, during 2020–2021 [29]. Weifang City in Shandong Province has indicated that the rates were 128.59, 137.65, and 100.84 during 2020–2023 [30]. Weifang City in Shandong Province has shown that the proportion of simultaneous vaccination is far lower than that of the two vaccines administered separately, which indirectly indicates that parents still have concerns about vaccination.

The WHO [31,32], Emilie [33], and Louise [34] have all indicated that parental concerns about adverse events following immunization (AEFI) are one of the main reasons for hesitancy regarding childhood vaccinations. Moreover, MMR is the NIP vaccine [35]. Possible reasons for choosing separate vaccination may include parents’ perception that the MMR vaccine causes “immune overload” in 8-month-old children and their concern that their children’s immune systems are “defective” [36]. Additionally, parents are worried about the potential pain, adverse reactions, and risk of serious illness associated with simultaneous vaccination, as well as its impact on children’s immune systems [37].

Guangzhou is located in the southeast coastal region of China and is one of the most economically developed cities in the country, with a permanent population of about 18.82 million and a vaccination volume of about 10 million [38]. There is also a relatively well-developed AEFI surveillance system. In 2020, Guangzhou implemented the policy of administering the first dose of the MMR vaccine to children aged 8 months. Therefore, the safety analysis after vaccination plays a crucial role in improving the quality of vaccination services and alleviating the concerns of recipients. This study retrospectively evaluated the adverse reaction reporting incidence rates for the simultaneous and separate administration of JEV-L and MMR among 8-month-old children in Guangzhou from 2020 to 2023. It preliminarily evaluated the safety of the concurrent administration of JEV-L and MMR, providing evidence to support public perceptions of the safety of simultaneous vaccination, thereby enhancing public confidence in concurrent vaccination.

## 2. Materials and Methods

### 2.1. Data Sources

Data were sourced from the Chinese National AEFI Information System (CNAEFIS). This is a passive surveillance system where the public or guardians can voluntarily report AEFIs. Cases are gathered by local County-level Center for Disease Control and Prevention (CDC), which is responsible for completing AEFI Case Reporting Cards and submitting the data to the CNAEFIS [39]. In addition, the vaccine data were obtained from the Guangdong Province Vaccine Distribution and Vaccination Management Information System, which covers 11 districts of Guangzhou, namely Baiyun, Conghua, Panyu, Huadu, Huangpu, Haizhu, Liwan, Nansha, Yuexiu, Tianhe, and Zengcheng.

### 2.2. Data Extraction

In the “Children’s Immunization Schedule (2021 Edition)”, it is planned that children will receive the JEV-L and the MMR at the age of 8 months [35]. This study extracted AEFI data and vaccination data from 1 June 2020, to 31 December 2023. The cases reported in the CNAEFIS include the following information: demographic information (gender, birthday, place of residence, etc.), vaccination-related information (type of vaccine, dose number, date of inoculation, date of onset, reporting date, time interval between vaccination and onset, etc.), and clinical information (clinical symptoms, clinical diagnosis, etiological classification, severity, etc.). The vaccination data collected include the vaccination information of the entire population from 2020 to 2023, covering data such as birthday, date of inoculation, type of vaccine, dose number, etc. The inclusion criteria were children born after 1 October 2019, and/or vaccinated with JEV-L or MMR after 1 June 2020, meeting the starting month for vaccination (8 months old); the vaccination dose was the first dose.

### 2.3. AEFI Classification and Definitions

In accordance with “National Suspected Vaccination Abnormal Response Monitoring Program” (Health Affairs Control Fa [2010] No. 94), AEFI refers to the reaction or event that occurs after vaccination and is suspected to be related to the vaccination [40]. All AEFI should be investigated by a group of AEFI investigation and diagnosis experts composed of at least five professional clinicians and epidemiologists, with the exception of common adverse reactions that have a clear diagnosis (e.g., fever, redness, swelling, and induration). After investigation, diagnosis, and analysis, the AEFI cases are classified into the following five categories according to the causes: adverse reactions (including common adverse reactions and rare adverse reactions), vaccine quality accidents, vaccination accidents, coincidences, and psychogenic reactions.

Common adverse reactions are the reactions that occur after vaccination and are caused by the inherent characteristics of the vaccine itself, which will only cause transient physiological dysfunction to the body, mainly including fever (classified into four levels: <37.0 °C, 37.1 °C to 37.5 °C, 37.6 °C to 38.5 °C, ≥38.6 °C), local redness (classified into three levels: none, 0.1 cm to 2.5 cm, ≥2.6 cm), swelling, and induration (classified into three levels: none, 0.1 cm to 2.5 cm, ≥2.6 cm) in this study. Rare adverse reactions refer to adverse reactions that damage the tissues, organs, and normal functions of recipients during or after vaccination with standard vaccines. Coincidences refer to when the recipient is in the incubation period or prodromal stage of a certain disease at the time of vaccination, and the disease coincidentally occurs after vaccination. According to the vaccine instructions, we have observed that both can elicit common reactions such as pain, fever, and rash. However, we have noticed that the more characteristic adverse reactions such as mild swelling of the parotid or salivary glands and arthritis following MMR may be related to the composition and mechanism of action of the MMR.

The simultaneous vaccination of JEV-L and MMR refers to the same recipient receiving both vaccines subcutaneously at different sites on the outer side of the upper arm, below the deltoid muscle attachment, within the same calendar day, and not mixed in the same syringe for injection. MMR separate vaccination refers to the administration of only MMR vaccine within a calendar day; JEV-L separate vaccination refers to the administration of only JEV-L vaccine within a calendar day. If two or more injectable attenuated live vaccines are not administered simultaneously, they should be administered with an interval of no less than 28 days.

### 2.4. Database Cleaning and Data Record Matching

During the data processing, we first removed records with logical errors in gender, outliers, and duplicates from the vaccination database. Subsequently, we matched the records from the vaccination database with those from the AEFI database based on unique identification codes, gender, date of birth, and vaccination dates. We carefully verified whether the vaccination information in the AEFI database matched that in the vaccination database, ultimately obtaining the database for analysis. Additionally, the two separate vaccination groups were matched based on unique identification codes, gender, date of birth, and vaccination dates to create a database for children who received the two vaccines separately on different calendar days.

### 2.5. Statistical Analysis

Microsoft Office Excel 2021, R software version 4.4.2, and SPSS software version 25.0 were used for data organization and statistical analysis. For categorical data (such as gender, vaccination season, and age distribution), the chi-square (χ^2^) test was used, with *p* < 0.05 indicating a statistically significant difference. The AEFI reporting rates for simultaneous administration of JEV-L and MMR, JEV-L alone, and MMR alone were calculated. The AEFI reporting rate for vaccines (per 100,000) is calculated as the number of cases with AEFI after receiving the vaccine divided by the total number of vaccine doses administered, multiplied by 100,000 doses. We used a univariate Poisson regression model to calculate the relative risk (RR) of adverse reactions and the 95% confidence interval (CI).

### 2.6. Ethics Statement

Before conducting this study, the study was conducted in accordance with the Declaration of Helsinki and approved by the Ethics and Research Committees of Guangzhou CDC (GZCDC-ECHR-2024P0174, approval date: 8 October 2024). Informed consent was obtained from all participants involved in this study.

## 3. Results

### 3.1. General Situation

**Figure 1:**
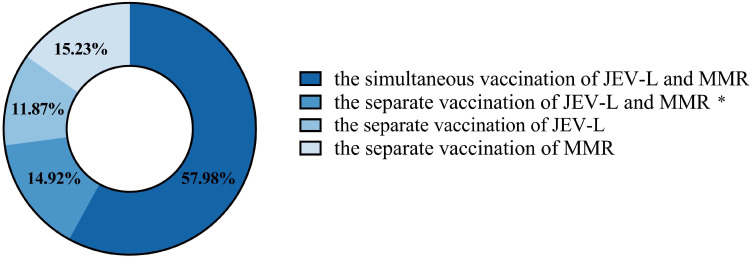
The distribution of children in Guangzhou who received JEV-L and MMR vaccines simultaneously or separately. *: The separate vaccination of JEV-L and MMR refers to children receiving JEV-L and MMR vaccines on two different calendar days, respectively, with an interval of 28 days or more.

In Guangzhou, a total of 214,238 doses of the first dose of the JEV-L vaccine were administered to children. There were 464,009 doses where the first dose of JEV-L was administered simultaneously with the first dose of MMR. A total of 241,150 doses of the first dose of MMR vaccine were administered. A total of 24 cases of AEFI were reported following JEV-L vaccination, with a total reporting rate (per 100,000 doses, the same below) of 11.20. A total of 246 cases of AEFI were reported following the simultaneous administration of JEV-L and MMR, with a reporting rate of 53.02. A total of 147 cases of AEFI were reported following the administration of MMR alone, with a reporting rate of 60.96. In Guangzhou, a total of 799,423 children were vaccinated, of whom 57.98% received simultaneous administration of JEV-L and MMR. A total of 14.92% of the children were vaccinated with JEV-L and MMR separately on different calendar days. A total of 11.87% of the children were vaccinated with JEV-L separately, and 15.23% of the children were vaccinated with MMR separately (Figure 1).

### 3.2. AEFI Incidence Characteristics

Between 2020 and 2023, the AEFI reporting incidence rate for the JEV-L vaccine administered alone in male children was 12.35. For the simultaneous vaccination of JEV-L and MMR, the rate was 52.49, and for the MMR administered alone, the rate was 64.26. The AEFI reporting incidence rates for female children were 9.91, 53.60, and 57.34, respectively. For children aged 8 to 11 months, the AEFI reporting incidence rates were 12.32, 54.37, and 60.48, respectively. For children aged 12 months and above, the rates were 5.60, 7.48, and 64.18, respectively (Table 1).

### 3.3. Time Interval of Adverse Reactions and Resolution Status

The time interval from vaccination to the occurrence of adverse reactions in children was within 24 h. The proportions of adverse reactions occurring within 24 h for JEV-L separate vaccination, the simultaneous vaccination of JEV-L and MMR, and MMR separate vaccination were 91.67% (22/24), 76.42% (188/246), and 56.46% (83/147), respectively.

In the JEV-L separate group, the outcomes were as follows: 13 cases (54.17%) improved, and 11 cases (45.83%) recovered completely. In the MMR separate group, the outcomes were as follows: 51 cases (34.69%) improved, 82 cases (55.78%) recovered completely, 13 cases (8.84%) required treatment, and 1 case had an unknown outcome. In the simultaneous vaccination of JEV-L and MMR, the outcomes were as follows: 98 cases (39.84%) improved, 127 cases (51.63%) recovered completely, 19 cases (7.72%) required treatment, 1 case worsened, and 1 case had an unknown outcome.

### 3.4. Adverse Reaction by Season

In terms of season, the summer season had higher vaccination doses and AEFI reporting incidence rates compared to other seasons, with rates of 12.81, 70.60, and 70.02 per 100,000 doses, respectively. In terms of the distribution of AEFI incidence by season, there was no significant difference between the group receiving JEV-L and MMR simultaneously and the other two groups (χ^2^ = 5.840, *p* = 0.120; χ^2^ = 5.519, *p* = 0.138). In terms of the reporting incidence rates across different seasons, there was a significant difference between the simultaneous vaccination of JEV-L and MMR and the JEV-L separate group, but no significant difference compared to the MMR separate group (Table 2).

### 3.5. AEFI Clinical Symptom Distribution

In the classification of AEFI, the proportions of common reactions were 87.50% (21/24), 88.21% (217/246), and 89.80% (132/147), respectively. The reporting rates were 9.80, 46.77, and 54.74, respectively. The reporting rates of rare adverse reactions were 0.93, 3.88, and 2.90, respectively. The reporting rates of coincidences were 0.47, 2.16, and 2.90, respectively. After vaccination with JEV alone, JEV-L and MMR simultaneously, and MMR alone, the total incidence rates of fever were 6.07, 26.94, and 29.86, respectively. The reporting incidence rates for fever ≥38.6 °C were 4.20, 16.16, and 17.83, respectively. For redness >2.5 cm, the rates were 0, 0.65, and 1.24, respectively; and for induration >2.5 cm, the rates were 0, 0.22, and 0.83, respectively.

For common adverse reactions, there was a significant difference between the simultaneous vaccination group and the JEV-L separate group (RR = 4.771, 95% CI = 3.048, 7.647) but no significant difference compared to the MMR separate group (RR = 0.854, 95% CI = 0.688, 1.060). For rare adverse reactions and coincidences, there was no significant difference between the simultaneous vaccination group and the other two groups (Table 3). For fever after vaccination, there was a significant difference between the simultaneous vaccination group and the JEV-L separate group (RR = 9.696, 95% CI = 1.304, 72.085; RR = 4.463, 95% CI = 1.359, 14.651; RR = 3.848, 95% CI = 1.927, 7.683) but no significant difference compared to the MMR separate group. For the occurrence of redness and induration in the simultaneous vaccination group, there was no significant difference compared to the other two groups (Table 3).

### 3.6. Distribution of AEFI Clinical Diagnoses

Among allergic reactions, there was a significant difference in the incidence of allergic rash between the simultaneous vaccination group and the JEV-L separate group (RR = 7.110, 95% CI = 3.750, 13.479). There was no significant difference in other clinical diagnoses (Table 3).

## 4. Discussion

Through matching in this study, it was found that 57.98% of children received JEV-L and MMR simultaneously. Compared with the percentage of doses in Weifang City, Shandong Province, more parents in Guangzhou accepted simultaneous vaccination [30]. This may be related to the policy implemented in 2019 that allowed multiple vaccines to be administered simultaneously [15], which in turn led to high compliance with the mandatory vaccination policy in 2020. A study in Shanghai showed that even if parents were hesitant about vaccination, they were still willing to accept the required vaccines according to the government’s recommended schedule [41]. In European studies, 68% of infant parents in Belgium and 60% in the United Kingdom were in favor of two injections during a single visit [42,43]. Similarly, in a multinational survey conducted in 2012, 70% of parents were most satisfied with two injections per visit as officially recommended or as suggested by providers [44]. Moreover, the attitudes of doctors towards simultaneous vaccination may influence parents’ acceptance. Studies have shown that the rational application of nursing guidelines can effectively improve compliance. Guangzhou has launched the Guangzhou Immunization Appointment System, which provides parents with relevant videos. The content includes the functions of vaccines, adverse reactions, and preventive measures, allowing parents to have a comprehensive understanding of the vaccines their children need to receive and thus improving compliance.

The fact that nearly half of the parents did not receive simultaneous vaccination indirectly indicates that there is still hesitation among parents regarding simultaneous vaccination for children at the age of 8 months. A study has shown that the process of maintaining public trust in vaccines is highly variable and depends on a thorough understanding of the community and its socioeconomic status, previous experiences, the opinions of those they trust (and distrust), as well as their understanding of the risks and benefits of vaccines compared to the diseases they prevent [45]. We should pay particular attention to enhancing parents’ confidence in simultaneous vaccination [46] and their understanding of the severity of the diseases.

In this study, by analyzing the vaccination data and AEFI data in Guangzhou from 2020 to 2023, it was found that the adverse reaction reporting rate in the simultaneous vaccination group was between that of the two separate vaccination groups. Meanwhile, the overall reporting rate of adverse reactions in the two separate vaccination groups was slightly lower than the national reporting rates of adverse reactions for 2021 and 2022 [47]. The incidence rates of general reactions and abnormal reactions were also lower compared to the national level. This discrepancy may be attributed to the inconsistent sensitivity of AEFI surveillance systems across different provinces and the varying criteria for the inclusion of subjects. The subjects in this study were all aged 8 months or older and had received their vaccinations after 1 June 2020. Additionally, the vaccine doses administered were the first doses. Currently, there are limited domestic studies on the passive surveillance of the simultaneous administration of these two vaccines. Weifang City in Shandong Province [30] and the province of Shandong [29] have both reported that the reporting rates of common and rare adverse reactions for the simultaneous vaccination group fall between the reporting rates of the two separate groups, which is consistent with the results of this study. In this study, the risk of AEFI in the simultaneous vaccination group was not significantly different from that in the group vaccinated with MMR alone, either for general reactions or for abnormal reactions. This finding is consistent with other studies on simultaneous vaccination [29,30,48,49]. The incidence rates observed in this study were lower than those reported in national surveillance data for JEV-L and MMR vaccinations in 2021 and 2022 [47]. However, when compared with the JEV-L separate group, there was a significant difference in the incidence of common reactions. This suggests that the simultaneous vaccination of JEV-L and MMR may increase the risk of common reactions compared to JEV-L alone. However, current monitoring indicates that the risk of common reactions with simultaneous vaccination does not increase compared to the MMR separate group.

We found that in the seasonal distribution of AEFI occurrences, summer was the primary season for all three groups, which is consistent with the results reported in Weifang City [30]. Additionally, research has shown that, apart from specific genetic and non-genetic host factors, seasonality is also an important environmental factor influencing various immune responses [50]. For example, a study by Lu Zhigang found that the number of peripheral blood T lymphocytes in humans is significantly higher in summer than in winter [51]. Moreover, the subjects included in the study were all vaccinated after June 2020, which may be the reason for the higher number of cases in the summer. There were more AEFI cases reported in the summer, which correspondingly led to a higher reporting rate. This phenomenon may be due to the fact that people wear more revealing clothing in the summer, exposing their arms. As a result, parents or caregivers may pay more attention to symptoms at the injection site and detect and report them in a timely manner. In terms of the reporting incidence rates across different seasons, there was no significant difference between the MMR separate group and the simultaneous vaccination group. However, a significant difference was observed between the JEV-L separate group and the simultaneous vaccination group. This indicates that simultaneous administration did not increase the risk of adverse reactions compared to the MMR separate group.

This study focused on the adverse reactions following the simultaneous administration of JEV-L and MMR in Guangzhou from 2020 to 2023. It was found that the main adverse reactions associated with the simultaneous administration of JEV-L and MMR, as well as with the separate administration of JEV-L and MMR, were fever and mild allergic rashes, which are consistent with the adverse reaction results from Shandong Province [29], Guangdong Province [52], and the post-marketing surveillance of JEV-L [19] and post-marketing adverse reactions of MMR globally [20]. The reporting rates of fever in the JEV-L separate group, the simultaneous vaccination of JEV-L and MMR, and the MMR separate group were lower than the national reporting rates of high fever following JEV-L and MMR vaccination for 2021 and 2022 [47] (13.11 and 15.93 for JEV-L; 21.72 and 31.01 for MMR). The incidence rate of fever in the MMR separate group is also lower than the incidence rate during the 32 years after MMR was marketed [20]. The reporting incidence rates for allergic rashes are higher than the national rates reported [47] (1.24 and 1.27 for JEV-L; 7.40 and 6.38 for MMR). The incidence rate of rash in the MMR alone group is lower than the incidence rate during the 32 years after MMR was marketed (46.3) [20]. The reason for the higher incidence in Guangzhou may be related to the city’s well-established monitoring process and the higher education level of parents. In addition, the degree of access to vaccine-related information and the level of vaccine trust may lead parents to believe that their young children have “immune deficiencies” [36,53,54], resulting in higher levels of anxiety and concern and thus a greater likelihood of reporting adverse reactions.

Compared with the other two groups, the simultaneous vaccination group did not show significant differences in the reporting incidence rates of local redness and induration. This indicates that simultaneous vaccination of JEV-L and MMR in 8-month-old children does not increase the risk of redness and induration. Additionally, there were no significant differences in the incidence rates of rare adverse reactions such as maculopapular rash, urticaria, and thrombocytopenic purpura between simultaneous vaccination and separate administration of JEV-L and MMR. This suggests that simultaneous vaccination of JEV-L and MMR in 8-month-old children does not increase the risk of these rare adverse reactions. Some studies have suggested that the MMR vaccine is currently the only vaccine for which a cause–effect relationship with immune thrombocytopenia [55]. Additionally, the attributable risk of developing idiopathic thrombocytopenic purpura (ITP) within 6 weeks after MMR vaccination is 1 in 25,000 vaccine doses (95% CI = 21,300 to 89,400) [56]. However, since ITP is relatively rare, further observation and analysis are needed to determine the safety differences between the two vaccination methods.

This study shows that adverse reactions to JEV-L and MMR, whether administered simultaneously or separately, mostly occur within 24 h after vaccination. This is basically consistent with the reports from the national level [47], Shandong Province [29], and Nanshan District of Shenzhen [57]. This suggests that medical staff and parents of children should pay attention to the 30 min on-site observation after vaccination. AEFI events should be reported, or medical attention should be sought promptly to ensure the timely management of adverse reactions.

The limitations of this study include the use of a passive reporting system to analyze vaccine safety. First, there are certainly differences in AEFI monitoring reports, disease diagnostic capabilities, and investigation capabilities between different regions. We made every effort to ensure that all monitors received standardized training and could guarantee the accuracy of the data. Secondly, the professional knowledge of doctors in vaccination clinics and the awareness level of guardians may lead to differences in the recognition of vaccine adverse reaction symptoms due to subjectivity. Thirdly, as a passive reporting system, vaccine recipients are likely to overlook the reporting of mild injection-site reactions, which can lead to an underestimation of the incidence rate of AEFIs. In future studies, we will try to use a combination of active and passive surveillance to collect data as much as possible.

## 5. Conclusions

In summary, the AEFI reporting incidence rate of the first dose of the simultaneous vaccination of JEV-L and MMR in 8-month-old children in Guangzhou is between the reporting incidence rates of the two separate groups. Compared with MMR separate vaccination, the simultaneous vaccination of JEV-L and MMR does not increase the risk of adverse reactions.

## Figures and Tables

**Table 1:** Distribution of AEFI reporting incidence rates for simultaneous and separate vaccination of JEV-L and MMR in children in Guangzhou.

Variable	JEV-L		JEV-L+MMR		MMR	
	Doses	*n* (Rate *)	Doses	*n* (Rate)	Doses	*n* (Rate)
Sex						
Male	113,361	14 (12.35)	243,873	128 (52.49)	126,050	81 (64.26)
Female	100,877	10 (9.91)	220,136	118 (53.60)	115,100	66 (57.34)
Age (months)						
8–11	178,528	22 (12.32)	450,633	245 (54.37)	209,988	127 (60.48)
≥12	35,710	2 (5.60)	13,376	1 (7.48)	31,162	20 (64.18)
Interval # (h)						
0–5	214,238	12 (5.60)	464,009	114 (24.57)	241,150	47 (19.49)
6–10	214,238	7 (3.27)	464,009	50 (10.78)	241,150	25 (10.37)
10–23	214,238	3 (1.40)	464,009	24 (5.17)	241,150	11 (4.56)
24–48	214,238	2 (0.93)	464,009	24 (5.17)	241,150	11 (4.56)
>48	214,238	0 (0.00)	464,009	34 (7.33)	241,150	53 (21.98)
Total	214,238	24 (11.20)	464,009	246 (53.02)	241,150	147 (60.96)

*: Reporting incidence rare/100,000 doses; #: Interval refers to the time interval between the occurrence of an adverse reaction after vaccination.

**Table 2:** Seasonal characteristics of JEV-L and MMR vaccination in 8-month-old children in Guangzhou.

Seasons	JEV-L (214,238)			JEV-L+MMR (464,009)			MMR (241,150)			JEV-L+MMR vs. VS JEV-L RR (95%CI)	JEV-L+MMR vs. VS MMR RR (95%CI)
	Doses	*n* (Rate *)	Percentage (%)	Doses	*n* (Rate)	Percentage (%)	Doses	*n* (Rate)	Percentage (%)		
Spring (3–5 months)	41,136	2 (4.86)	8.33	104,401	69 (66.09)	8.33	54,511	30 (55.03)	20.41	13.594 (3.332, 55.454)	1.201 (0.782, 1.844)
Summer (6–8 months)	78,038	10 (12.81)	41.67	141,641	100 (70.60)	41.67	78,548	55 (70.02)	37.41	5.51 (2.876, 10.556)	1.008 (0.725, 1.401)
Autumn (9–11 months)	56,622	7 (12.36)	29.17	123,479	50 (40.49)	29.17	65,741	39 (59.32)	26.53	3.275 (1.485, 7.224)	0.683 (0.449, 1.038)
Winter (12–the next year 2 months)	38,442	5 (13.01)	20.83	94,488	27 (28.58)	20.83	42,350	23 (54.31)	15.65	2.197 (0.846, 5.705)	0.526 (0.302, 0.917)

*: Reporting incidence rare/100,000 doses.

**Table 3:** Adverse reaction reporting incidence rates for simultaneous and separate vaccination of JEV-L and MMR in 8-month-old children in Guangzhou.

Variable	JEV-L (214,238)		JEV-L+MMR (464,009)		MMR (241,150)		JEV-L+MMR vs. JEV-L RR (95%CI)	JEV-L+MMR vs. MMR RR (95%CI)
	n	Rates *	n	Rates	n	Rates		
AEFI types								
Common adverse reactions	21	9.80	217	46.77	132	54.74	4.771 (3.048, 7.467)	0.854 (0.688, 1.060)
Rare adverse reactions	2	0.93	18	3.88	7	2.90	4.155 (0.964, 17.907)	1.336 (0.558, 3.199)
Coincidences	1	0.47	10	2.16	7	2.90	4.617 (0.591, 36.068)	0.742 (0.282, 1.949)
Fever (°C)								
37.1–37.5	1	0.47	21	4.53	7	2.90	9.696 (1.304, 72.085)	1.559 (0.663, 3.667)
37.6–38.5	3	1.40	29	6.25	22	9.12	4.463 (1.359, 14.651)	0.685 (0.394, 1.192)
≥38.6	9	4.20	75	16.16	43	17.83	3.848 (1.927, 7.683)	0.906 (0.623, 1.318)
Redness								
≤2.5 cm	1	0.47	3	0.65	4	1.66	1.385 (0.144, 13.315)	0.390 (0.087, 1.743)
>2.5 cm	0	0.00	3	0.65	3	1.24	/	0.520 (0.105, 2.576)
Induration								
≤2.5 cm	0	0.00	1	0.22	1	0.41	/	0.520 (0.033, 8.314)
>2.5 cm	0	0.00	1	0.22	2	0.83	/	0.260 (0.024, 2.867)
Clinical diagnoses								
Maculopapule	1	0.47	4	0.86	1	0.41	1.847 (0.206, 16.526)	2.079 (0.232, 18.601)
Urticaria	1	0.47	12	2.59	7	2.90	5.541 (0.720, 42.615)	0.891 (0.351, 2.263)
Measles-like and scarlatiform rash	0	0.00	2	0.43	1	0.41	/	1.039 (0.094, 11.459)
Allergic rash	10	4.67	154	33.19	80	33.17	7.110 (3.750, 13.479)	1.000 (0.763, 1.310)
Thrombocytopenic purpura	0	0.00	6	1.29	3	1.24	/	1.039 (0.260, 4.155)
Other #	1	0.47	2	0.43	10	4.15	0.923 (0.084, 10.18)	2.599 (0.569, 11.862)

*: Reporting incidence rare/100,000 doses; #: convulsions, seizures, viral infection and coincidences.

## Data Availability

Data cannot be shared openly due to the protection of patient information and privacy. Access to the data is subject to approval and a data-sharing agreement with the Guangzhou CDC.
